# Adjuvants Enhance the Induction of Germinal Center and Antibody Secreting Cells in Spleen and Their Persistence in Bone Marrow of Neonatal Mice

**DOI:** 10.3389/fimmu.2019.02214

**Published:** 2019-09-26

**Authors:** Audur Anna Aradottir Pind, Magdalena Dubik, Sigrun Thorsdottir, Andreas Meinke, Ali M. Harandi, Jan Holmgren, Giuseppe Del Giudice, Ingileif Jonsdottir, Stefania P. Bjarnarson

**Affiliations:** ^1^Department of Immunology, Landspitali, The National University Hospital of Iceland, Reykjavik, Iceland; ^2^Faculty of Medicine, School of Health Sciences, University of Iceland, Reykjavik, Iceland; ^3^Valneva Austria GmbH, Vienna, Austria; ^4^Department of Microbiology and Immunology, Institute of Biomedicine, Sahlgrenska Academy, University of Gothenburg, Gothenburg, Sweden; ^5^Vaccine Evaluation Center, BC Children's Hospital Research Institute, The University of British Columbia, Vancouver, BC, Canada; ^6^University of Gothenburg Vaccine Research Institute (GUVAX), Department of Microbiology and Immunology, University of Gothenburg, Gothenburg, Sweden; ^7^GSK Vaccines, Siena, Italy; ^8^deCODE Genetics/Amgen, Reykjavík, Iceland

**Keywords:** vaccination, neonate, adjuvant, germinal center, antibody-secreting cell persistence, spleen, bone marrow, protective antibodies

## Abstract

Immaturity of the immune system contributes to poor vaccine responses in early life. Germinal center (GC) activation is limited due to poorly developed follicular dendritic cells (FDC), causing generation of few antibody-secreting cells (ASCs) with limited survival and transient antibody responses. Herein, we compared the potential of five adjuvants, namely LT-K63, mmCT, MF59, IC31, and alum to overcome limitations of the neonatal immune system and to enhance and prolong responses of neonatal mice to a pneumococcal conjugate vaccine Pnc1-TT. The adjuvants LT-K63, mmCT, MF59, and IC31 significantly enhanced GC formation and FDC maturation in neonatal mice when co-administered with Pnc1-TT. This enhanced GC induction correlated with significantly enhanced vaccine-specific ASCs by LT-K63, mmCT, and MF59 in spleen 14 days after immunization. Furthermore, mmCT, MF59, and IC31 prolonged the induction of vaccine-specific ASCs in spleen and increased their persistence in bone marrow up to 9 weeks after immunization, as previously shown for LT-K63. Accordingly, serum Abs persisted above protective levels against pneumococcal bacteremia and pneumonia. In contrast, alum only enhanced the primary induction of vaccine-specific IgG Abs, which was transient. Our comparative study demonstrated that, in contrast to alum, LT-K63, mmCT, MF59, and IC31 can overcome limitations of the neonatal immune system and enhance both induction and persistence of protective immune response when administered with Pnc1-TT. These adjuvants are promising candidates for early life vaccination.

## Introduction

Vaccines against infectious diseases have a major impact on human health, preventing each year 2–3 millions deaths worldwide (1). In 2017 5.4 million children under 5 years died, mostly in developing countries, whereof 2.5 million deaths occurred in the first month of life (2). Infectious diseases caused a large part of these deaths, many of which are vaccine-preventable (2). These numbers emphasize the need for effective approaches to limit infections and preventable deaths in early life. Vaccination is the most efficacious way to fight infection and eliminate pathogens.

The neonatal immune system is immature resulting in increased vulnerability to infections and poor vaccine-induced immune responses in early life. Although protective vaccines against many pathogens are available, the vaccine-induced antibody (Ab) responses wane after 6–9 months, and hence multiple vaccinations are essential to maintain protection and immunological memory (3).

The immune system of 1 week old mice and responses to various antigens (Ag) correspond well to those of human neonates, both in terms of Ab responses and T cell function, and those of 3 weeks old mice correspond to human infants (4, 5). Induction of germinal centers (GCs) is delayed in spleen of human neonates (6, 7), in agreement with the limited follicular dendritic cell (FDC) maturation and GC induction in neonatal mice (8, 9). Furthermore, the delayed induction of T follicular helper cells (Tfh) is circumvented by adjuvants like MF59 in infant but not neonatal mice (10). Similarily, Tfh response was limited in naive children (11), but MF59 increased IFNγ^+^ and TNFα^+^IL-2^+^ secreting CD4^+^ T cells in human infants vaccinated with tri-valent influenza vaccine (12). We have shown that immunogenicity of pneumococcal conjugate vaccine is highly age dependent and plain pneumococcal polysaccharide (PPS) is only immunogenic in adult mice but not infant or neontal mice, reproducing the main features of human infant immune responses to native capsular PPS and pneumococcal conjugate vaccines, as well as the relative protective efficacy of those vaccines. In our mouse model of pneumococcal vaccination and infection LT-K63 enhanced the response to Pnc1-TT but not to the polysaccharide in neonatal and infant mice, in line with its effect on the FCDs and GCs which play a key role in response to T cell-dependent (TD) Ags but not T cell-independent (TI) Ags (9, 13–15).

Neonatal activation of GCs is limited leading to formation of few plasmablasts whose survival in bone marrow (BM) is reduced, resulting in low and transient Ab responses (16, 17). In GCs, B cells undergo clonal expansion and affinity maturation, and consequently the differentiation into Ab-producing plasmablasts and plasma cells (PC) or memory cells. FDCs can bind and present Ag-Ab immune complexes to B cells via complement receptors or Fc receptors (18, 19). FDCs together with Tfh orchestrate the GC reaction. The crosstalk between GC B cells and Tfh influences B cell survival, proliferation and differentiation and promotes development of memory B cells and long-lived PCs (20). Interestingly, there is a preferential differentiation of memory B cells rather than PCs in neonates [reviewed in (4)]. Most plasmablasts emerging from neonatal GCs migrate efficiently to the BM, but do not persist. Instead they undergo apoptosis resulting in rapid decline of serum Abs during early life (16, 21). This is explained by insufficient survival signals, mainly APRIL, from the BM niches, leaving the plasmablasts unable to differentiate into long-lived PCs and persist in the BM niche (16, 21, 22).

Adjuvants are immune stimulating agents and are central components of vaccines that enhance both magnitude and duration of immune responses and may modulate the nature of the responses. Alum was the only adjuvant included in licensed human infant vaccines until MF59 was included in H1N1 influenza vaccine licensed in Europe in 2009 for vaccination from 6 months of age (23, 24). The adjuvant LT-K63 is a non-toxic, genetically modified derivative of heat labile enterotoxin from *E. coli*, that interacts with a variety of immunocompetent cells and mediates immunomodulation associated with adjuvanticity. LT-K63 was originally developed as a mucosal adjuvant but also elicited strong adjuvanticity when given parenterally [reviewed in (13, 14, 25)]. We showed for the first time that LT-K63 could stimulate GC formation and enhance maturation of FDC network in neonatal mice. Immunization of neonatal mice with Pnc1-TT and LT-K63 resulted in increased Ab responses and increased specific ASCs in spleen and survival in BM, leading to persistence of protective Abs against pneumococcal lung infection and bacteremia (9, 13, 14). We have previously shown that Pnc1-TT with LT-K63 or CpG-ODN induced comparable protective Ab levels and had comparable effects on neonatal dendritic cells, B cells and T cells (26–28). However, only LT-K63 improved T helper cell type 1 (Th1) activation (26, 28), increased TNF expression associated with enhanced FDC maturation, GC induction and prolonged persistence of ACSs in spleen and BM [(9) and unpublished data]. The clinical development of LT-K63 was stopped due to transient facial nerve paralysis observed in two vaccinees following intranasal delivery (29). Recently, the ability of the adjuvant, CAF01 to induce GC reaction in neonatal mice was reported (30). There is an unmet need of developing safe and potent adjuvants that can overcome the limitations of early life immunity (31). Further, a better understanding of the mode of action of existing adjuvants may contribute to development of improved neonatal and infant vaccination strategies (32).

In search for an optimal neonatal adjuvant we evaluated the potential adjuvant effect of four adjuvants with different modes of action, namely mmCT, MF59, IC31, and CTB-CpG, on various neonatal immune parameters, with emphasis on GC induction, FDC maturation, and the generation of ASCs in the spleen, their migration to and long-term survival in the BM, translating into persistent protective Ab levels. Alum and LT-K63 were used for comparison. mmCT is a novel, non-toxic multiple-mutant of cholera toxin (CT) (33) that in addition to increasing IgG and IgA Ab responses to co-administered antigen and strong IFN-γ and IL-17A T cell responses in mice (34), it has been shown to promote human Th17 responses (35) through activation of the classical NF-κB pathway of monocytes (36). MF59, a squalene-based oil-in-water emulsion, was well tolerated and enhanced protective efficacy of influenza vaccination in infants and young children (37), meeting all the European licensure criteria (38) and has been licensed for use in children from 6 months with seasonal influenza vaccine (23). IC31, which combines two immunomodulatory compounds; an antibacterial peptide KLK (11-mer cationic peptide KLKL_5_KLK) and a TLR9 agonist, a synthetic oligodeoxynucleotide (ODN1a) without a CpG motif, has been shown to rapidly enhance protective humoral responses in neonates when combined with Pnc1-TT (39). CTB-CpG is a potent novel adjuvant that combines the effects of CpG and the non-toxic B subunit of cholera toxin (40, 41). Our comparative study shows that LT-K63, mmCT, MF59 and IC31 can overcome limitations of the neonatal immune system and enhance both induction and persistence of protective immune responses when administered with Pnc1-TT.

## Materials and Methods

### Mice

Adult NMRI mice were purchased from Taconic (Skensved, Denmark) and allowed to adapt for 1 week before mating. They were kept in microisolator cages with free access to commercial food pellets and water, and housed under standardized conditions at ArcticLAS vivarium facility (Reykjavík, Iceland), with regulated daylight, humidity and temperature. Breeding cages were checked daily for new births, and pups kept with their mothers until weaning at 4 weeks of age. This study was carried out in accordance with the Act No. 55/2013 on animal welfare and regulations 460/2017 on protection of animals used for scientific research. The protocol was approved by the Experimental Animal Committee of Iceland (license no. 2015-10-01).

### Vaccine and Adjuvants

PPS of serotype 1 (PPS1) conjugated to TT (Pnc1-TT) (42) was provided by Sanofi Pasteur (Marcy l'Etoile, France). LT-K63 and MF59 were produced by Novartis Vaccines and Diagnostics, (now GSK Vaccines, Siena, Italy) as described previously (43, 44). mmCT and CTB-CpG were produced as described elsewhere (33, 45). IC31 was produced by Intercell AG, (now Valneva, Vienna, Austria) as described (46). Aluminum hydroxide (Alhydrogel) was purchased from Brenntag Biosector A/S (Ballerup, Denmark).

### Immunizations

Neonatal (1 week old) mice (8 mice/group, except 3–4 mice/group in a screening experiment) were immunized subcutaneously (s.c.) at base of tail with 0.5 μg of Pnc1-TT alone or mixed with the adjuvants LT-K63 (5 μg/mouse), mmCT (2 μg/mouse), MF59 (50% of injected volume/mouse), IC31 (50 nmol KLK and 2 nmol ODN1a/mouse), CTB-CpG (5 μg/mouse), or Alum (0.48% aluminum hydroxide per 1 μg of protein/mouse) in 50 μl of saline. We compared s.c. immunizations at scapular girdle as used in earlier studies (9, 13, 14, 26, 39) with s.c. immunization at base of tail and observed no difference between the routes, as comparable GC induction was observed in the spleen along with comparable vaccine-specific ASC in spleen and BM and Abs in serum (Figure S1), indicating that independent of the s.c. immunization site and draining LNs, the efficiency of Ag transport to the spleen is similar.

Blood was obtained from the tail vein at various time points after priming; serum was isolated and stored at −20°C. Spleens were removed and half was mounted in OCT, snap frozen, and kept at −70°C; the other half was used to enumerate PPS1 and TT-specific ASCs. BM was collected for enumeration of specific ASCs.

### ELISA

PPS1- and TT-specific Abs (IgG) were measured by ELISA (47). Microtiter plates (MaxiSorp; Nunc AS, Roskilde, Denmark) were coated with 5 μg PPS1/ml (American Type Culture Collection, Rockville, MD) in PBS for 5 h at 37°C or 5 μg TT (Sanofi Pasteur) per milliliter in 0.10 M carbonate buffer (pH 9.6) overnight at 4°C and blocked with PBS-Tween 20 and 1% BSA (Sigma). Serum samples and standard were neutralized by cell wall polysaccharides (Statens Serum Institute, Copenhagen, Denmark). Neutralized sera and standard in duplicates were serially diluted (3-fold dilutions) and were incubated at room temperature for 2 h, followed by HRP goat anti-mouse Ab (Southern Biotechnology Associates, Birmingham, AL). The reaction was developed by 3,3′,5,5′-tetramethylbenzidine-substrate (Kirkegaard & Perry Laboratories, Gaithersburg, MD), stopped with 0.18 M H_2_SO_4_, and read at 450 nm in Titertek Multiscan Plus MK II spectrophotometer (ICN Flow Laboratories, Irvine, U.K.). Results were expressed as mean log ELISA units (EU)/ml ± SD, calculated from a standard curve from at least two different dilutions with coefficient of variation between duplicates and dilutions below 20% (47).

### ELISPOT

PPS1- and TT-specific ASC were enumerated by ELISPOT, as previously described (9, 48). MultiScreen High protein binding immobilon-P membrane plates (Millipore Corporation, Bedford, MA) were coated with 20 μg/ml PPS1 or 10 μg/ml TT overnight at 37°C, blocked with complete RPMI 1640 (Life Technologies BRL, Life Technologies, Paisley, U.K.). Duplicates of cells from spleen and BM were tested in four three-fold dilutions starting with 1 × 10^7^ cells in 100 μL in complete RPMI 1640 per well (9, 48) and incubated for 5 h at 37°C, washed and incubated with ALP-goat anti-mouse IgG (Southern Biotechnology Associates) overnight at 4°C, and developed by 5-bromo-4-chloro-3-indolylphosphate and NBT in AP development buffer (Bio-Rad Labs, Hercules, CA). The number of spots, each representing a cell secreting specific Abs, were counted by ELISPOT reader ImmunoSpot^®^ S6 ULTIMATE using ImmunoSpot^®^ SOFTWARE (Cellular Technology Limited (CTL) Europe, Bonn, Germany).

### Immunofluorescent Staining of Tissue Sections

Spleens were frozen in Tissue-Tek OCT (Sakura, Zouterwoude, the Netherlands) and cut into 7 μm cryosections at 2 levels, starting 1,750 μm into the tissue; the levels were separated by 210 μm, fixed in acetone for 10 min, and stored at −70°C. Two sections/spleen (one from each level) were stained with fluorescent labeled IgM-FITC (BD Pharmingen) to visualize the follicles, and biotinylated peanut agglutinin (PNA)-bio (Vector Laboratories, Burlingame, CA) to label dark-zone B cells, a good marker for active GC reaction. Adjacent sections from both levels of spleen were stained with primary monoclonal Abs (mAbs) FDC-M2 (biotinylated) (AMS Biotechnology Limited, Oxfordshire, U.K) for mature FDCs and MOMA-1 (AbD Serotec, Düsseldorf, Germany) for metallophilic marginal macrophages, respectively. Primary Abs were incubated at RT for 30 min. The sections were then washed in PBS for 2 × 5 min prior to incubation with APC Streptavidin (BD Biosciences, Stockholm, Sweden) at RT for another 30 min and sections washed again as before. DAPI (Invitrogen, Eugene, OR) was used for nuclear counterstaining. The sections were photographed with a digital camera (AXIOCAM; Zeiss) in a microscope (Zeiss) equipped with X10 and X40 20 objectives and AxioImaging Software (Birkerod, Denmark) for light and three-color immunofluorescence. Areas of PNA- and FDC-M2-positive staining were measured from all pictures using the AxioImaging Software.

### Statistical Analysis

Mann-Whitney U test was used for comparison between groups. Results were considered significant at *p* < 0.05. All statistical analyses were carried out using Graphpad Prism 7.03 (GraphPad Software, La Jolla, CA).

## Results

### Screening and Selection of Adjuvants

First, we screened for potential effects of the adjuvants mmCT, MF59, IC31, and CTB-CpG on the neonatal immune response compared to the previously established effects of LT-K63 (9) on the induction of GC reaction and enhanced Ab response in neonates. Neonatal mice were immunized s.c. with Pnc1-TT, with/without adjuvants. GC activation and maturation of FDC in spleen was assessed 14 days after immunization and vaccine-specific Abs in serum measured 14 and 35 days after immunization. Immunofluorescent staining revealed that mmCT and MF59 enhanced GC formation in spleen to a comparable degree as LT-K63 (Figure S2). Accordingly, mmCT, MF59, and IC31 yielded comparable Ab responses as that induced by LT-K63, while CTB-CpG did not increase Ab responses compared to Pnc1-TT alone (Figure S3). Based on those results (Figures S2, S3) mmCT, MF59, and IC31 were selected for further evaluation of their effects on primary B cell induction and humoral response compared to those of LT-K63 and alum. As CTB-CpG seemed to neither enhance the GC activation nor vaccine-specific Abs it was not included in further experiments.

### LT-K63, mmCT, MF59, and IC31 Enhance Induction of GCs in Neonatal Mice

**Figure 1:**
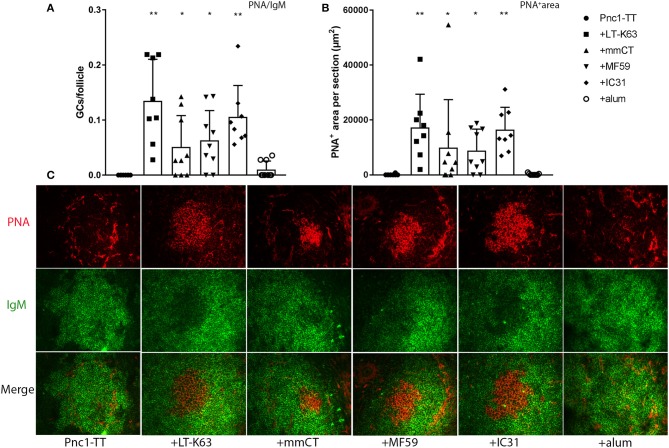
Effects of adjuvants on germinal center induction. Spleen sections were stained with fluorescent Abs for PNA and IgM 14 days after immunization of neonatal mice with Pnc1-TT with/without adjuvants LT-K63, mmCT, MF59, IC31, or alum. PNA/IgM ratio represents activated GCs in relation to total number of follicles **(A)** and PNA^+^ area represents total area of positive PNA staining per section **(B)**. Representative immunofluorescense staining pattern for PNA (red) and IgM (green) of each group is shown in **(C)**. Results are expressed as mean + SD in 8–9 mice per group and statistics done using Mann–Whitney *U*-test where adjuvant groups were compared to vaccine only group: ^*^p < 0.05, ^**^p < 0.001.

Neonatal mice were immunized with Pnc1-TT with or without LT-K63, mmCT, MF59, IC31, or alum. Spleen sections were stained with anti-IgM and PNA to evaluate the adjuvant effects on GC induction 14 days after immunization. IgM staining detects naïve B cells in the follicles and PNA staining identifies highly proliferating B cell centroblasts located in the dark zone of the GCs. Compared to Pnc1-TT alone, all adjuvants except alum significantly enhanced GC formation, demonstrated by increased PNA/IgM ratio (Figure 1A, Figure S4, and Table S1), increased total area of PNA^+^ staining and average size of GCs (Figure 1B, Figure S4E, and Table S1). PNA staining patterns of representative individuals for each group (Figure 1C) showed a clear difference between mice immunized with Pnc1-TT with LT-K63, mmCT, MF59, or IC31 compared to Pnc1-TT only. The PNA staining was intense in the adjuvant groups with distinctive formation of PNA^+^ follicles. In contrast, mice that received only Pnc1-TT had few, faint, small and poorly formed PNA^+^ GCs. The Pnc1-TT + alum group also showed weak GC staining, similar to that of mice immunized with Pnc1-TT alone. Therefore, in contrast with alum, neonatal immunization with LT-K63, mmCT, MF59, or IC31 combined with Pnc1-TT could overcome the early life limitations of GC induction.

### The Impact of LT-K63, mmCT, MF59, and IC31 on FDC Maturation and Metallophilic Macrophage Migration Into Splenic B Cell Follicles in Neonates

**Figure 2:**
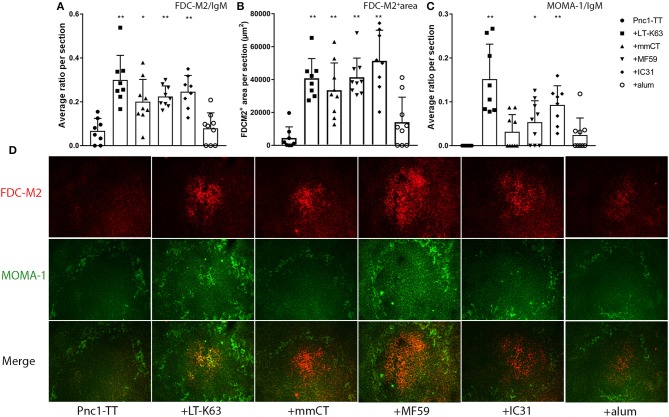
Effects of adjuvants on follicular dendritic cell maturation and migration of marginal metallophillic macrophages into follicles. Spleen sections were stained with fluorescent Abs for FDC-M2 and MOMA-1 14 days after immunization of neonatal mice with Pnc1-TT with/without adjuvants LT-K63, mmCT, MF59, IC31, or alum. FDC-M2/IgM ratio represents number of fully developed FDC networks in relation to total follicles **(A)**, FDC-M2^+^ area represents total area of positive FDC-M2 staining per section **(B)**, and MOMA-1/IgM ratio represents count of MOMA-1^+^MMM migration into follicles in relation to total follicles **(C)**. Representative immunofluorescense staining pattern for FDC-M2 (red) and MOMA-1 (green) of each group is shown in **(D)**. Results are expressed as mean + SD in 8–9 mice per group and statistics done using Mann–Whitney *U*-test where adjuvant groups were compared to vaccine only group: ^*^p < 0.05, ^**^p < 0.001.

To explore whether adjuvant-enhanced GC formation was mediated by accelerated FDC network maturation, spleen sections obtained 14 days after priming with Pnc1-TT with/without adjuvant were stained with anti-FDC-M2 that identifies complement fragment C4 on mature immune-complex-bearing FDCs (49). To assess if the adjuvants induce migration of immune complex/Ag-bearing marginal metallophilic macrophages (MMM) into activated follicles, spleen sections were stained for MOMA-1 (CD169), a lectin-like receptor expressed on different macrophages populations including splenic MMMs and subcapsular macrophages of LNs (50, 51). MOMA-1^+^ macrophages show migratory properties in response to bacterial stimuli like LPS (52). LT-K63, mmCT, MF59, and IC31 significantly enhanced maturation of FDC-M2^+^ FDC clusters in spleen, shown by increased FDC-M2/IgM ratio, compared to vaccine alone (Figure 2A and Table S1). All these adjuvants also significantly enhanced the total area of FDC-M2^+^ staining (Figure 2B and Table S1) and average size of FDC-M2^+^ FDC clusters (Figure S1F). However, alum had no effect on number or size of FDC-M2^+^ FDC clusters in spleen. MOMA-1 staining revealed that LT-K63, MF59, and IC31 significantly enhanced the migration of MMMs into follicles where they co-localized with FDC-M2^+^ FDCs, demonstrated by MOMA-1/IgM ratio, compared to vaccine alone (Figure 2C and Table S1), whereas the effects of mmCT and alum on MOMA-1/IgM ratio were not significant. Immunofluorescence staining patterns of representative individuals from each group (Figure 2D) show that neonatal mice immunized with Pnc1-TT with LT-K63, mmCT, MF59, or IC31 had well formed FDC-M2^+^ clusters, with intense and even staining, while LT-K63 had the strongest effect on MMM migration into the activated follicles. In contrast, the Pnc1-TT + alum group showed weak staining of FDC-M2^+^ clusters, similar to mice that only received Pnc1-TT. Taken together, the adjuvants LT-K63, mmCT, MF59, and IC31 accelerated maturation of FDC-M2^+^ FDC clusters, in contrast to alum where no effect was observed.

### mmCT and MF59 Enhance the Induction of Vaccine-Specific ASCs in Spleen

**Figure 3:**
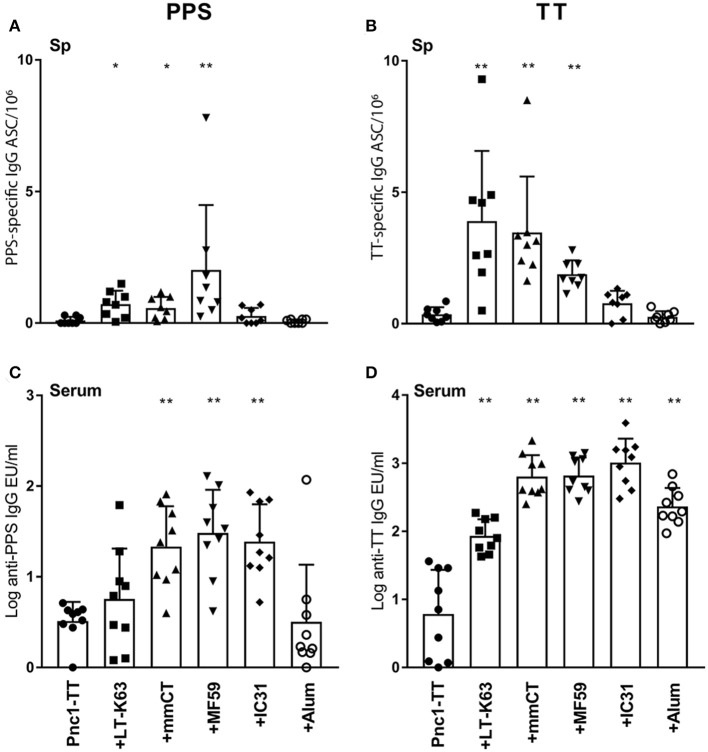
Adjuvants can induce enhanced induction of vaccine-specific antibody-secreting cells in spleen and increased induction of vaccine-specific antibodies in serum. PPS1-specific **(A,C)** and TT-specific **(B,D)** ASC in spleen **(A,B)** and Abs in serum **(C,D)** 14 days after immunization of neonatal mice with Pnc1-TT with or without the adjuvants LT-K63, MF59, IC31, mmCT, or alum. Results are expressed as number of spots/10^6^ cells (mean + SD) or IgG levels (mean EU/ml + SD) in 8–9 mice per group. Statistical difference was calculated using Mann–Whitney *U*-test where adjuvant groups were compared to vaccine only group: ^*^p < 0.05, ^**^p < 0.0001.

Next we investigated whether enhanced GC formation, evident by PNA staining, correlated with enhanced primary induction of vaccine-specific ASCs in spleen and consequently, enhanced vaccine-specific Abs in serum. LT-K63, mmCT, and MF59 induced significantly higher number of PPS1- and TT-specific ASCs than Pnc1-TT alone, in spleen 14 days after immunization (Figures 3A,B and Table S3). All adjuvants significantly enhanced TT-specific Abs in serum compared to Pnc1-TT alone (Figure 3D) and mmCT, MF59, and IC31 significantly enhanced PPS1-specific Abs compared to vaccine alone (Figure 3C and Table S2). Interestingly, immunization with Pnc1-TT with MF59 or IC31 prolonged the induction of PPS1-specific ASCs in spleen reflected by higher number of PPS1-specific ASCs in spleen 6 weeks after immunization compared to mice that received only Pnc1-TT (Figure S5 and Table S3). Mice immunized with Pnc1-TT with MF59 still had significantly increased numbers of PPS1-specific ASCs in spleen up to 9 weeks later (Figure S5 and Table S3). Increased numbers of TT-specific ASCs in spleen were also observed 9 weeks after immunization with Pnc1-TT with mmCT, MF59, or IC31 (Figure S5 and Table S3).

### mmCT, MF59, and IC31 Increase the Persistence of Vaccine-Specific ASCs in Bone Marrow

**Figure 4:**
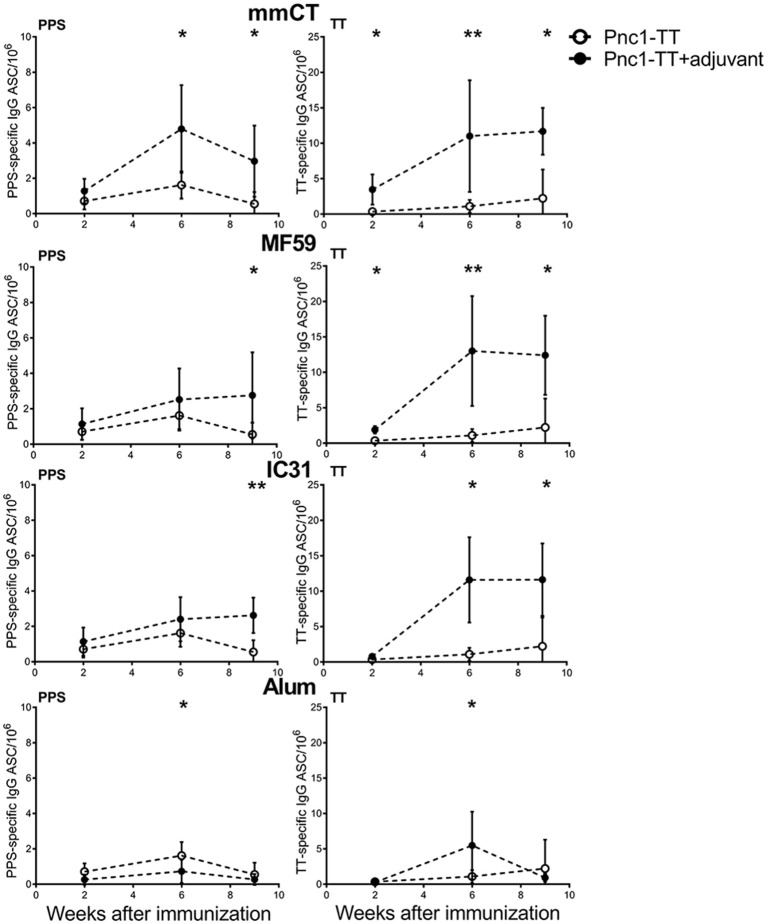
Effects of adjuvants on migration and persistence of vaccine-specific antibody-secreting cells in bone marrow. PPS1-specific (left panel) and TT-specific (right panel) ASC in bone marrow 2, 6, and 9 weeks after priming with Pnc1-TT with/without the adjuvants mmCT, MF59, IC31, or alum. Results are expressed as number of spots/10^6^ cells (mean ± SD) in 8 mice per group (except *n* = 7 for MF59 at week 6). Statistical difference was calculated using Mann–Whitney *U*-test where adjuvant groups were compared to vaccine only group: ^*^p ≤ 0.05, ^**^p ≤ 0.001.

We have previously shown that LT-K63 enhances induction of primary vaccine-specific ASCs and prolongs their persistence in spleen and BM (9). Thus, to investigate the effect of mmCT, MF59, IC31, and alum on migration and long-term persistence of vaccine-specific ASCs in BM they were enumerated 2, 6, and 9 weeks after one neonatal immunization. Mice immunized as neonates with Pnc1-TT with mmCT or MF59 had already significantly increased number of TT-specific ASCs in BM 2 weeks after immunization and mmCT also significantly increased PPS1-specific ASC numbers in BM 6 weeks after immunization (Figure 4 and Table S4). All adjuvants, including alum, enhanced the numbers of TT-specific ASCs in BM 6 weeks after administration with Pnc1-TT, compared to vaccine alone (Figure 4 and Table S4). Mice immunized with Pnc1-TT with mmCT, MF59, or IC31 still had increased numbers of PPS1- and TT-specific ASCs in BM 9 weeks after immunization, whereas there was no difference between mice immunized with Pnc1-TT only and those that received Pnc1-TT with alum. This shows that alum-enhanced immune responses in neonatal mice are more transient than those induced by the other adjuvants (Figure 4 and Table S4).

### mmCT, MF59, and IC31 Increase the Persistence of Vaccine-Specific Abs in Serum

**Figure 5:**
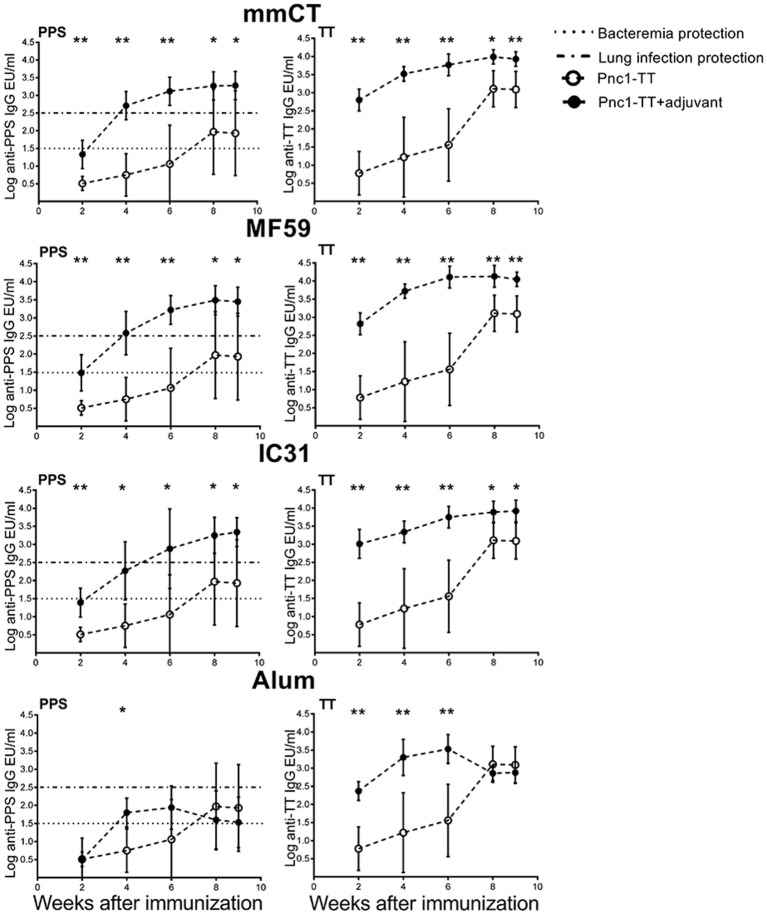
Kinetics of vaccine-specific IgG antibody response after neonatal immunization. PPS1- (left panel) and TT-specific (right panel) serum Abs 2, 4, 6, 8, and 9 weeks after immunization of neonatal mice with Pnc1-TT with/without the adjuvants mmCT, MF59, IC31, or alum. Results are expressed as IgG levels (EU/ml + SD). The dotted line (log EU/ml = 1.5) represents a sufficient antibody titer to protect against serotype 1 pneumococcal bacteremia while the dashed line (log EU/ml = 2.5) represents an antibody titer sufficient for protection against serotype 1 pneumococcal lung infection. Statistical difference was calculated using Mann-Whitney *U*-test where adjuvant groups were compared to vaccine only group: ^*^p ≤ 0.05, ^**^p ≤ 0.001.

Next we assessed the adjuvant effects on Ab responses, both primary induction and long-term persistence of Abs up to 9 weeks after immunization. All the adjuvants significantly enhanced TT-specific Abs over the time period while all except alum significantly enhanced PPS1-specific Abs (Figure 5 and Table S2). Even though alum enhanced the primary induction of vaccine-specific IgG Abs, the responses were transient and 8–9 weeks after immunization there was no difference between serum Abs in mice immunized with Pnc1-TT only and Pnc1-TT with alum, in contrast to the persisting increase in Ab levels induced by the other adjuvants (Table S2). The protective PPS1-specific IgG Ab levels of log 1.5 EU/ml and log 2.5 EU/ml against pneumococcal bacteremia and lung infections, respectively, are well established in this model of neonatal, infant and adult mice when challenged intranasally with *S. pneumoniae* of serotype 1 (13–15, 28, 39, 53, 54). All mice that were immunized as neonates with one dose of Pnc1-TT with mmCT, MF59, or IC31 reached anti-PPS1 IgG levels close to 2 log EU/ml above protective levels against pneumococcal bacteremia and close to 1 log EU/ml above protective Ab levels against lung infection, that persisted for 9 weeks after immunization (Figure 5 and Table S5). Taken together, our results demonstrate a superiority of the novel adjuvants tested here compared to alum to induce persistent protective Ab responses at early age compared to alum.

## Discussion

In this study, we compared the potential of mmCT, MF59, and IC31, to overcome limitations of the neonatal immune system and induce robust and long-lasting responses to immunization. LT-K63 and alum were used for comparison. LT-K63, MF59, and IC31 have previously been shown to enhance some neonatal immune responses (9, 10, 28, 30, 55) while effects of mmCT on neonatal immunity have not been reported. We found that all the adjuvants except alum, enhanced neonatal immune responses, both initiation and persistence. Our results show little benefits of alum in early life immunization with a pneumococcal conjugate vaccine.

LT-K63, mmCT, MF59, and IC31 all enhanced GC induction after neonatal immunization with Pnc1-TT, shown by PNA staining of spleen sections. Until now only LT-K63 (9), and recently CAF01 (30) have been shown to overcome delayed induction and elicit fully established GCs in neonates where HA/CAF01 (30) and Pnc1-TT/LT-K63 (9) elicited organized and well-structured GCs upon neonatal immunization. In the current study, IC31 given with Pnc1-TT accelerated the maturation of FDCs and induced formation of GCs. This contrasts results from neonatal immunization with HA/IC31 where GC formation was not observed (30) and neonatal immunization with IC31 and pneumococcal proteins, which did neither accelerate FDC maturation nor induce GC formation [(28) and unpublished data]. Likewise, no GCs were observed in dLNs 10 days after immunization of neonatal mice with HA/MF59 (10) even though they were observed in spleen 14 days post immunization with Pnc1-TT + MF59 in this study. Furthermore, in our study GCs were almost absent when alum was administered with Pnc1-TT, while GCs were detected in spleen 14 days after i.p. immunization of neonatal mice with alum-adsorbed TT in a different study (8). Thus, it seems that not only the adjuvant but the doses and combination of antigen and adjuvant, and possibly the immunization route, lymphoid tissue, and mouse strain studied, affect whether adult-like induction of GCs is observed after neonatal immunization. In addition, the difference in the antigen used may not be trivial, a conjugate vaccine can behave differently than an influenza hemagglutinin. The polysaccharide is not an inherent component in front of the carrier, and may play a role acting directly on B cells.

All the adjuvants tested in this study, except alum, accelerated the maturation of FDC-M2^+^ FDC clusters. FDC-M2 Ab reacts with murine FDCs and specifically recognizes an activation form of the complement fragment C4 (49). In GCs, FDCs retain and present immune complexes to B cells through complement- and Fc-receptors (18). We have previously shown that LT-K63 can restore delayed maturation of FDCs and induce adult-like FDC-M2^+^ FDC clusters when administered to neonatal mice with Pnc1-TT (9). LT-K63 is, until now, the only adjuvant that has been reported to overcome this delayed FDC maturation while potent adjuvants like CpG, which induce adult-like B- T- and dendritic cell responses have failed to do (8, 9). Although CAF01 can elicit fully established GCs in neonates (30) its effect on neonatal FDC-M2^+^ FDC maturation has not been reported. Others have proposed the role of FDC-M1^+^ FDCs (56, 57) and CR1 (56, 58) in the organization of B cell follicles and maintenance of GC in early life, whereas our previous work did not indicate that they were limiting factors for FDC network maturation (9). In agreement with our results, i.p. immunization of neonatal mice with TT adsorbed to AL(OH)_3_ did not accelerate maturation of FDC clusters (8).

Blood-borne Ags are primarily trapped in the marginal zone of the spleen. MZ B cells (59, 60) and MMMs have been shown to be crucial for trapping of particulate Ags (61) in spleen and subcapsular sinus macrophages in LNs (50, 51). MOMA-1^+^ macrophages are required for immune responses against TD and TI-2 Ags (62, 63). We observed increased migration of MOMA-1^+^ macrophages that co-localized with FDC-M2^+^ FDCs in mice immunized with Pnc1-TT with LT-K63, MF59, and IC31. This correlated with enhanced GC reaction and later increased frequencies of vaccine-specific Abs and ASCs. However, even though enhanced GC activation and increased frequencies of vaccine-specific ASCs were observed in mice immunized with Pnc1-TT and mmCT, this adjuvant did not enhance migration of MMMs into follicles. It is possible that day 14 after immunization is not optimal for evaluating and comparing the effects of all the adjuvants as they have different mechanisms of action and different kinetics.

Ab persistence is mediated by long-lived memory PCs that reside in specialized survival niches in the BM. It has been noted that most of the plasmablasts emerging from the GC in neonates migrate efficiently to the BM, but lack ability to persist. Instead they undergo apoptosis resulting in rapid decline of serum Abs during early life (16, 21). We found that mmCT, MF59, and IC31 all enhanced persistence of vaccine-specific ASCs in BM when administered with Pnc1-TT. As suspected, increased persistency of ASCs in BM coincided with increased levels of vaccine-specific IgG Abs. It is encouraging that only one dose of Pnc1-TT with the adjuvants mmCT, MF59, or IC31 in neonates was enough to induce protective Ab levels against both pneumococcal bacteremia and pneumonia (13, 53, 54) since one of the great challenges of neonatal immunization is the need of multiple vaccinations to induce and maintain protection and immunological memory (3). Likewise, a single dose of HA with adjuvants CAF01 (30) or Advax™ (64) administered to 7 days old mice has been shown to be protective against influenza challenge.

We have shown the superiority of intranasal immunization with Pnc1-TT and LT-K63 in neonatal and infant mice compared to parenteral immunization, in terms of enhanced specific mucosal IgA and systemic IgG and protection from pneumococcal bacteremia and lung infection (13). mmCT is a potent mucosal adjuvant (33–36) and here we show its effect as a parenteral adjuvant in neonates. Potential advantage of mucosal immunization against encapsulated respiratory bacteria at early age needs to be further studied (65). However, parenteral vaccination can also be effective in inducing protective responses at mucosal sites, especially when formulated with mucosal trafficking adjuvants, able to alter expression of mucosal homing markers and tissue destination [reviewed in (66)].

There is still limited clinical information on the safety of vaccine adjuvants in newborns and, more in general, in infants. This is due to the slow process of vaccine development which requires cautious testing first in adults, then in adolescents, and finally in infants. Among the adjuvants tested in our study, the largest experience has been acquired with MF59-adjuvanted seasonal, pandemic, and avian influenza vaccines. MF59-adjuvanted seasonal influenza vaccine had a stronger efficacy as compared to licensed non-adjuvanted influenza vaccines in infants (37), it was more immunogenic than the non-adjuvanted vaccine in infants and toddlers and it exhibited an excellent safety profile (12, 37, 67). It must be added that cytokine profile induced by the MF59-adjuvanted vaccine in infants did not differ from that found in adults (i.e., a Th0/Th1-type profile), with no evidence of induction of Th2-type responses (12, 67). The same strong immunogenicity was also observed with the pandemic and with avian influenza vaccines (68). Another oil-in-water adjuvant, containing alpha-tocopherol, referred to as AS03 (not included in this study), was reported to be associated with the insurgence of narcolepsy when administered with pandemic A/H1N1 vaccine in young children (69). However, these allegations were never formally substantiated by experimental data (70). More studies will be required with other vaccine adjuvants to show their safety profile in young children and ultimately in infants.

Taken together, by comparing the effects of different adjuvants on GC reaction, ASC in spleen and BM and serum Abs in neonatal mice we have shown that in contrast to alum, the most widely used adjuvant in childhood vaccinations, LT-K63, mmCT, MF59, and IC31 all enhanced GC reaction and FDC maturation when administered with a pneumococcal conjugate vaccine. Furthermore, LT-K63, mmCT, MF59, and IC31 induced increased numbers of vaccine-specific ASCs that persisted in BM 9 weeks after immunization, which was reflected in increased vaccine-specific serum Abs persisting above protective levels against pneumococcal disease (13, 53, 54). This demonstrates that it is possible to achieve adult-like long-lived responses to neonatal vaccination by the use of selected adjuvants. These results warrant further investigation of mmCT, MF59, and IC31 as promising candidate adjuvants for early life vaccination.

## Data Availability

The datasets generated for this study are available on request to the corresponding author.

## Ethics Statement

This study was carried out in accordance with the Act No. 55/2013 on animal welfare and regulations 460/2017 on protection of animals used for scientific research. The protocol was approved by the Experimental Animal Committee of Iceland (license no. 2015-10-01).

## Author Contributions

AA, IJ, and SB conceived and designed the study, analyzed the data, and wrote the manuscript. IJ and SB supervised the study. AH, JH, AM, and GD provided material and expertise. AA, MD, ST, and SB performed the experiments. AA, AH, JH, AM, GD, IJ, and SB interpreted the results. All authors contributed to and approved the final version of the manuscript.

### Conflict of Interest Statement

GD is full-time employee and holds shares in the GSK group of companies. AM is an employee of Valneva Austria GmbH. The remaining authors declare that the research was conducted in the absence of any commercial or financial relationships that could be construed as a potential conflict of interest.
