# Supplementary material for: Adjuvants Enhance the Induction of Germinal Center and Antibody Secreting Cells in Spleen and Their Persistence in Bone Marrow of Neonatal Mice
Source: Front Immunol. 2019 Sep 26;10:2214. doi: 10.3389/fimmu.2019.02214 (PMC6775194; doi:10.3389/fimmu.2019.02214)
Supplement: Supplementary file 1 [file Presentation_1.pdf]

Supplementary Material

Adjuvants enhance the induction of germinal center and antibody secreting cells in spleen and their persistence in bone marrow of neonatal mice

Audur Anna Aradottir Pind, Magdalena Dubik, Sigrun Thorsdottir, Andreas Meinke, Ali M. Harandi, Jan Holmgren, Giuseppe Del Giudice, Ingileif Jonsdottir, Stefania P. Bjarnarson*

* **Correspondance:** Stefania P. Bjarnarson: stefbja@landspitali.is

1 Supplementary Figures and Tables

1.1 Supplementary Figures

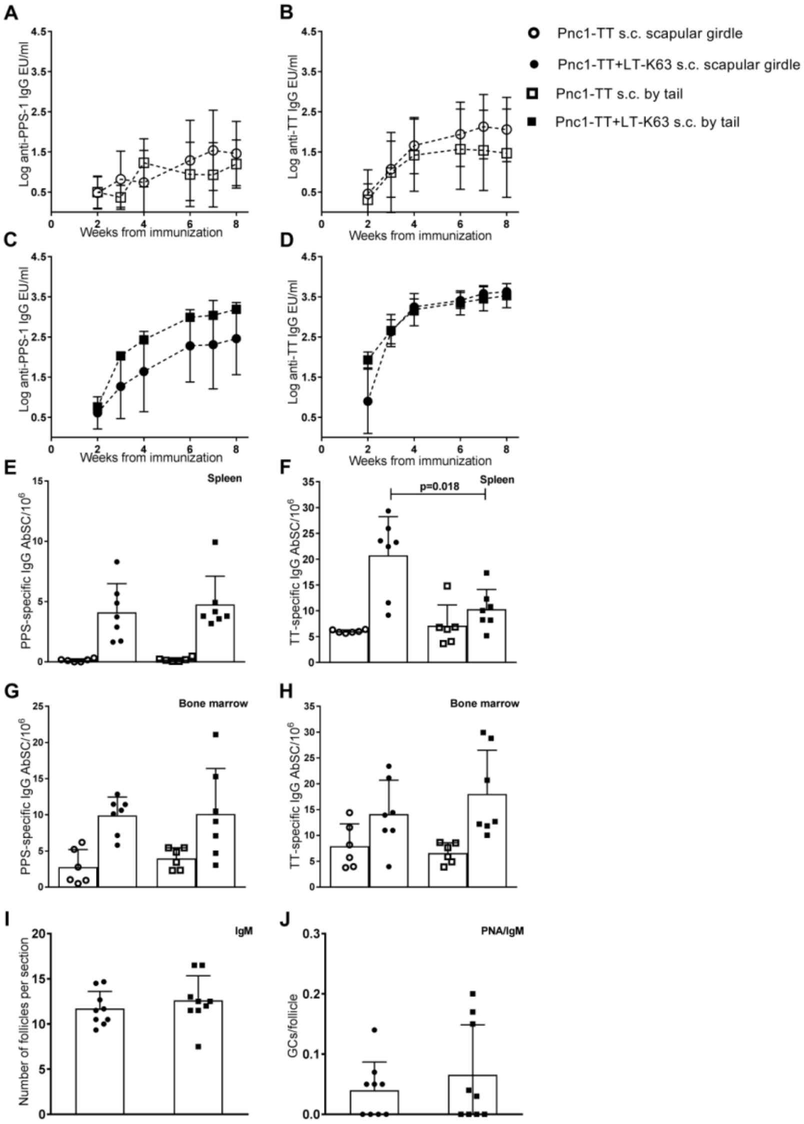

Supplementary Figure 1. Comparison of site of injection in scapular girdle vs. base of tail. Neonatal mice were immunized s.c. at scapular girdle (circle) or at base of tail (square) with Pnc1-TT (open circle/square) or Pnc1-TT with LT-K63 (filled circle/square). No difference was observed in vaccine-specific antibody kinetics between injection sites (A-D). Vaccine-specific antibody-secreting cells were enumerated 56 days after immunization in spleen (E-F) and bone marrow (G-H). Spleen sections of mice immunized with Pnc1-TT+LT-K63 at either scapular girdle or base of tail were cryosectioned 14 days after immunization and stained for IgM to enumerate follicles per section (I) and PNA to evaluate numbers of germinal centers per follicle (J). We concluded that the two different sites of injection yielded comparable results in regard to all immune parameters assessed. Results are expressed as mean + SD and statistical difference was calculated using Mann-Whitney U test.

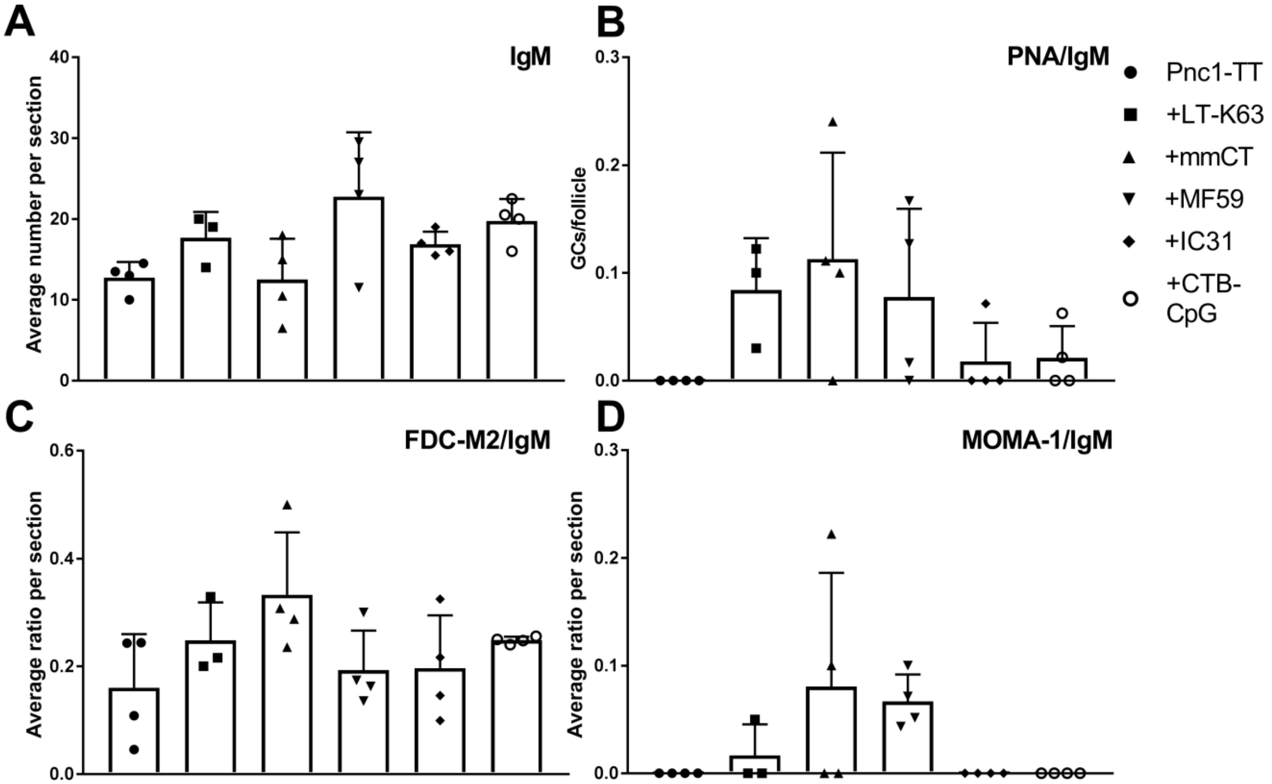

Supplementary Figure 2. Effects of adjuvants on germinal center induction, follicular dendritic cell maturation and migration of marginal metallophillic macrophages into follicles. Neonatal mice were immunized with Pnc1-TT w/wo adjuvants LT-K63, mmCT, MF59, IC31 or CTB-CpG. Spleen sections were stained with fluorescent antibodies for IgM, PNA, FDC-M2 and MOMA-1. IgM staining (A) represents the number of follicles per section, PNA/IgM ratio (B) represents activated GCs in relation to total number of follicles, FDC-M2/IgM ratio (C) represents number of fully developed FDC networks in relation to total follicles and MOMA-1/IgM ratio (D) represents number of MOMA-1 migration into follicles in relation to total follicles. Results are expressed as mean + SD in 3-4 mice per group.

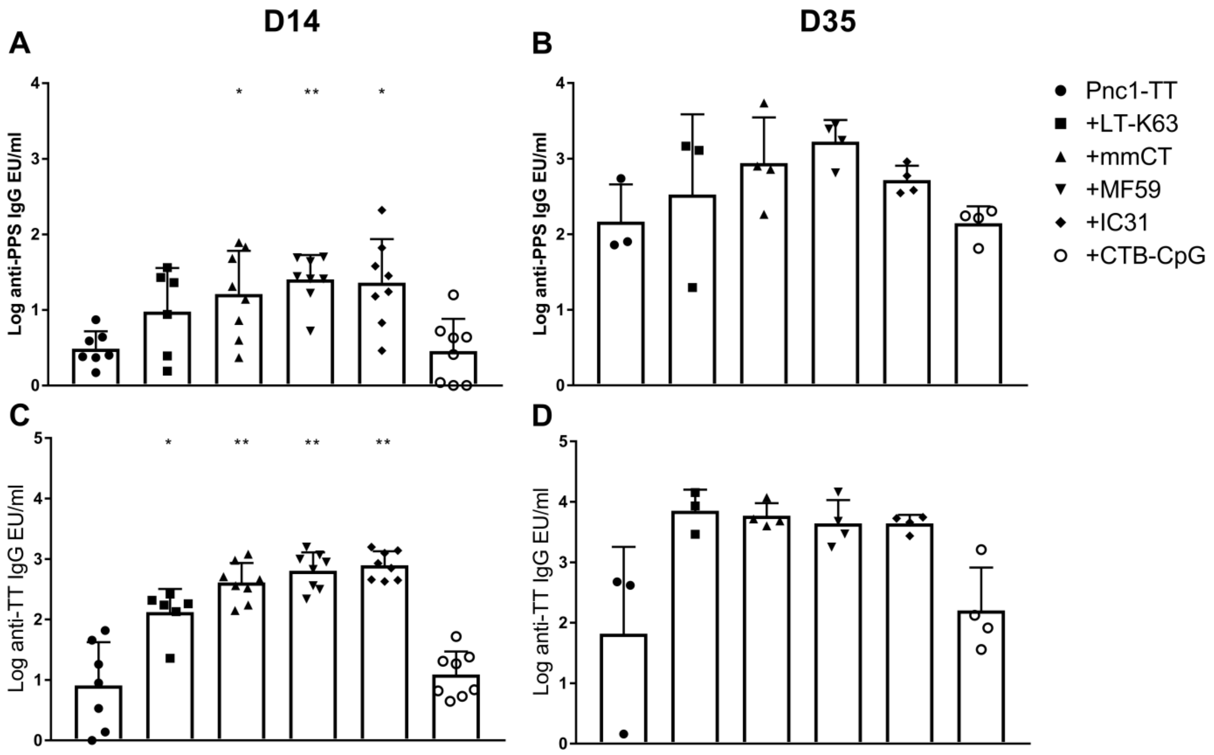

Supplementary Figure 3. Vaccine-specific antibodies to PPS1 and TT 14 and 35 days after immunization of neonates with Pnc1-TT with/without adjuvants LT-K63, mmCT, MF59, IC31 or CTB-CpG. The results are expressed as mean + SD of log EU/ml (n=6-8 per group for D14, n=3-4 per group for D35). Statistical difference was calculated using Mann-Whitney U test where adjuvant groups were compared to vaccine only group: *$p \leq 0.05$, **$p \leq 0.001$.

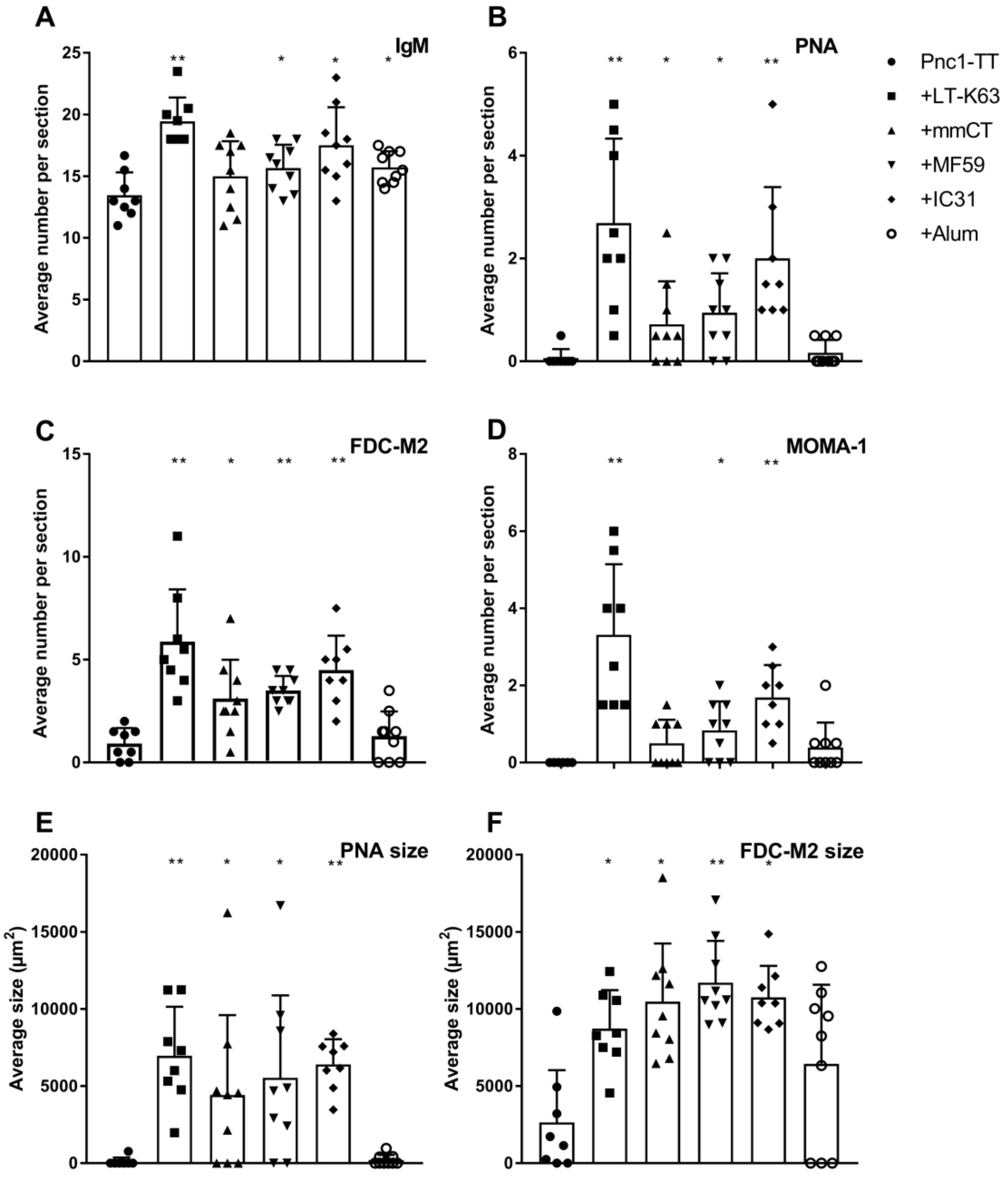

Supplementary Figure 4. Effects of adjuvants on germinal center induction, follicular dendritic cell maturation and migration of marginal metallophilic macrophages into follicles. Spleen sections were stained with fluorescent antibodies for PNA and IgM 14 days after immunization of neonatal mice with Pnc1-TT with/without adjuvants LT-K63, mmCT, MF59, IC31 or alum. IgM represents total number of follicles per section (A), PNA represents total number of activated follicles, germinal centers (GC), per section (B), FDC-M2 represents total number of matured FDC-$M2^+$ follicular dendritic cell (FDC) clusters per section (C), MOMA-1 represents migration of marginal metallophilic macrophages into follicles per section (D), PNA size represents average size of GCs per section (E) and FDC-M2 size represents average size of mature FDC-$M2^+$ FDC clusters per section (F). Results are expressed as mean + SD in 8-9 mice per group and statistics done using Mann-Whitney U test where adjuvant groups were compared to vaccine only group: *$p<0.05$, **$p<0.001$.

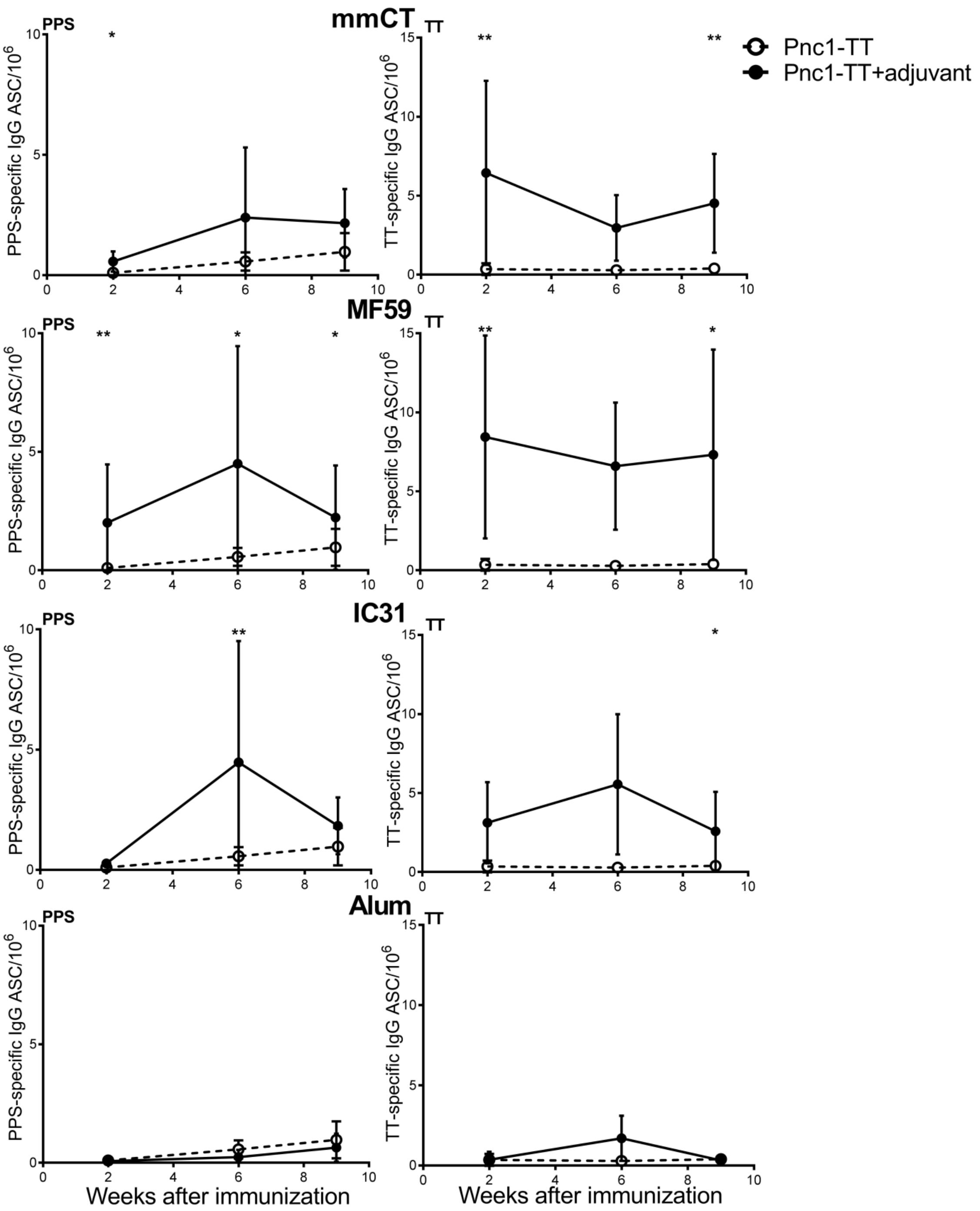

Supplement Figure 5. Effects of adjuvants on induction of vaccine-specific antibody-secreting cells in spleen. PPS-specific (left panel) and TT-specific (right panel) antibody-secreting cells in spleen 2, 6 and 9 weeks after priming with Pnc1-TT with/without the adjuvants mmCT, MF59, IC31 or alum. Results are expressed as number of spots/10^6 cells (mean ± SD) in 8 mice per group (except n=7 for MF59 at week 6). Statistical difference was calculated using Mann-Whitney U test where adjuvant groups were compared to vaccine only group: *p≤0,05, **p≤0,001.

1.2 Supplementary Tables

Supplementary Table 1. Results from immunofluoroscent stainings 14 days after priming with Pnc1-TT with/without adjuvants LT-K63, mmCT, MF59, IC31 or alum. Results are expressed as median values ± SD. Statistics are calculated by comparing adjuvant groups to Pnc1-TT group using Mann-Whitney U test and $p \leq 0.05$, shown in bold, was considered statistically significant.

	**Pnc1-TT**	**+LT-K63**		**+mmCT**		**+MF59**		**+IC31**		**+Alum**	
	Median ± SD	Median ± SD	p	Median ± SD	p	Median ± SD	p	Median ± SD	p	Median ± SD	p
**IgM count**	13.00 ± 1.75	18.75 ± 1.82	**0.0002**	15.50 ± 2.68	0.2649	16.00 ± 1.78	**0.0291**	17.50 ± 2.92	**0.0060**	15.50 ± 1.23	**0.0141**
**PNA+ count**	0 ± 0.18	2.25 ± 1.65	**0.0003**	0.50 ± 0.83	**0.0331**	1.00 ± 0.77	**0.0071**	1.5 ± 1.39	**0.0002**	0 ± 0.25	0.5765
**PNA+/IgM**	0 ± 0.01	0.12 ± 0.08	**0.0002**	0.03 ± 0.06	**0.0090**	0.05 ± 0.05	**0.0023**	0.09 ± 0.06	**0.0002**	0 ± 0.02	0.2059
**PNA$^+$ area (μm)**	0 ± 273	16144 ± 12083	**0.0002**	4551 ± 17491	**0.0146**	4833 ± 7810	**0.0037**	13791 ± 8187	**0.0002**	0 ± 339	0.5059
**GC average size (μm)**	0 ± 273	6655 ± 3184	**0.0002**	4442 ± 5179	**0.0146**	4691 ± 5355	**0.0037**	6681 ± 1625	**0.0002**	0 ±339	0.5059
**FDC-M2+ count**	0.92 ± 0.76	5.25 ± 2.55	**0.0002**	2.50 ± 1.88	**0.0033**	3.50 ± 0.71	**<0.0001**	4.50 ± 1.67	**0.0003**	1.50 ± 1.20	0.6456
**FDC-M2+/IgM**	0.07 ± 0.06	0.27 ± 0.11	**0.0002**	0.17 ± 0.10	**0.0037**	0.21 ± 0.05	**<0.0001**	0.26 ± 0.08	**0.0003**	0.09 ± 0.07	0.8124
**FDC-M2+ area (μm)**	1559 ± 6788	39879 ± 12006	**0.0002**	30989 ± 16569	**0.0003**	38471 ± 11659	**<0.0001**	50913 ± 18661	**0.0002**	11054 ± 15178	0.3143
**FDC average size (μm)**	1433 ± 3390	8353 ± 2487	**0.0470**	9557 ± 3778	**0.0016**	10593 ± 2711	**0.0003**	10381 ± 2049	**0.0011**	8249 ± 5139	0.2696
**MOMA-1+ count**	0 ± 0	3.25 ± 1.83	**0.0002**	0 ± 0.61	0.0824	1.00 ± 0.75	**0.0090**	1.75 ± 0.84	**0.0002**	0 ± 0.65	0.0824
**MOMA-1+/IgM**	0 ± 0	0.08 ± 0.04	**0.0002**	0 ± 0.04	0.0824	0.06 ± 0.05	**0.0090**	0.1 ± 0.04	**0.0002**	0 ± 0.04	0.0824

Supplementary Table 2. Vaccine-specific antibody responses 2, 4, 6, 8 and 9 weeks after priming with Pnc1-TT with/without LT-K63, mmCT, MF59, IC31 or alum. Results are expressed as median values of log EU/mL ± SD. Statistics are calculated by comparing adjuvant groups to Pnc1-TT group using Mann-Whitney U test and p≤0.05, shown in bold, was considered statistically significant.

	**Pnc1-TT**		**+mmCT**				**+MF59**				**+IC31**				**+Alum**			
	**PPS1-specific**	**TT-specific**	**PPS1-specific**		**TT-specific**		**PPS1-specific**		**TT-specific**		**PPS1-specific**		**TT-specific**		**PPS1-specific**		**TT-specific**	
**Weeks**	Median ± SD	Median ± SD	Median ± SD	p	Median ± SD	p	Median ± SD	p	Median ± SD	p	Median ± SD	p	Median ± SD	p	Median ± SD	p	Median ± SD	p
**2**	0.59 ± 0.21	0.64 ± 0.64	1.38 ± 0.45	**0.0005**	2.80 ± 0.33	**<0.0001**	1.53 ± 0.48	**0.0003**	2.69 ± 0.26	**<0.0001**	1.27 ± 0.41	**<0.0001**	3.14 ± 0.34	**<0.0001**	0.23 ± 0.63	0.2672	2.26 ± 0.28	**<0.0001**
**4**	0.62 ± 0.57	1.18 ± 1.05	2.81 ± 0.39	**0.0003**	3.56 ± 0.21	**0.0002**	2.86 ± 0.57	**0.0006**	3.70 ± 0.24	**0.0003**	2.66 ± 0.76	**0.0019**	3.38 ± 0.32	**0.0003**	1.75 ± 0.39	**0.0026**	3.43 ± 0.46	**0.0003**
**6**	0.59 ± 1.08	1.34 ± 1.02	3.24 ± 0.44	**0.0006**	3.77 ± 0.31	**0.0002**	3.45 ± 0.38	**0.0006**	4.21 ± 0.31	**0.0003**	3.37 ± 1.06	**0.0076**	3.74 ± 0.33	**0.0003**	1.80 ± 0.63	**0.083**	3.61 ± 0.45	**0.0006**
**8**	1.87 ± 1.19	3.00 ± 0.52	3.26 ± 0.39	**0.0139**	3.98 ± 0.22	**0.0016**	3.43 ± 0.35	**0.0028**	4.27 ± 0.33	**0.0005**	3.34 ± 0.46	**0.0155**	3.83 ± 0.31	**0.0055**	1.83 ± 0.78	0.4807	2.87 ± 0.23	0.1458
**9**	1.86 ± 1.21	2.97 ± 0.47	3.33 ± 0.42	**0.0152**	3.94 ± 0.18	**0.0007**	3.46 ± 0.40	**0.0078**	4.05 ± 0.22	**0.0003**	3.48 ± 0.37	**0.0152**	3.88 ± 0.25	**0.0014**	1.70 ± 0.72	0.5414	2.93 ± 0.28	0.4965

Supplementary Table 3. Vaccine-specific antibody-secreting cells in spleen 2, 4 and 9 weeks after priming with Pnc1-TT with/without mmCT, MF59, IC31 or alum. Results are expressed as median number of spots/10^6 cells ± SD. Statistics are calculated by comparing adjuvant groups to Pnc1-TT group using Mann-Whitney U test and $p \leq 0.05$, shown in bold, was considered statistically significant.

	**Pnc1-TT**		**+mmCT**				**+MF59**				**+IC31**				**+Alum**			
	**PPS1-specific**	**TT-specific**	**PPS1-specific**		**TT-specific**		**PPS1-specific**		**TT-specific**		**PPS1-specific**		**TT-specific**		**PPS1-specific**		**TT-specific**	
**Weeks**	Median ± SD	Median ± SD	Median ± SD	p	Median ± SD	p	Median ± SD	p	Median ± SD	p	Median ± SD	p	Median ± SD	p	Median ± SD	p	Median ± SD	p
**2**	0 ± 0.14	0.18 ± 0.38	0.53 ± 0.42	**0.011**	5.83 ± 4.93	**0.0002**	1.10 ± 2.47	**0.0006**	6.42 ± 7.88	**0.0002**	0.15 ± 0.31	0.2973	2.57 ± 2.03	0.0999	0.05 ± 0.08	0.8238	0.18 ± 0.51	0.4559
**6**	0.63 ± 0.38	0.14 ± 0.35	1.43 ± 2.92	0.0878	2.08 ± 2.86	0.9579	4.97 ± 2.36	**0.0019**	5.03 ± 4.03	0.0721	5.05 ± 2.56	**0.0006**	4.44 ± 3.42	**0.083**	0.14 ± 0.31	0.0870	1.74 ± 1.41	0.3832
**9**	0.78 ± 1.03	0.27 ± 0.47	2.13 ± 1.42	0.0876	3.13 ± 2.65	**0.0002**	2.20 ± 1.31	**0.0462**	6.66 ± 6.63	**0.0011**	1.18 ± 1.33	0.2455	2.50 ± 1.91	**0.0047**	0.39 ± 0.59	0.5737	0.31 ± 0.28	0.9581

Supplementary Table 4. Vaccine-specific antibody-secreting cells in bone marrow 2, 4 and 9 weeks after priming with Pnc1-TT with/without mmCT, MF59, IC31 or alum. Results are expressed as median number of spots/10^6 cells ± SD. Statistics are calculated by comparing adjuvant groups to Pnc1-TT group using Mann-Whitney U test and p≤0.05, shown in bold, was considered statistically significant.

	**Pnc1-TT**		**+mmCT**				**+MF59**				**+IC31**				**+Alum**			
	**PPS1-specific**	**TT-specific**	**PPS1-specific**		**TT-specific**		**PPS1-specific**		**TT-specific**		**PPS1-specific**		**TT-specific**		**PPS1-specific**		**TT-specific**	
**Weeks**	Median ± SD	Median ± SD	Median ± SD	p	Median ± SD	p	Median ± SD	p	Median ± SD	p	Median ± SD	p	Median ± SD	p	Median ± SD	p	Median ± SD	p
**2**	0.70 ± 0.47	0.30 ± 0.27	1.03 ± 0.69	0.1049	3.08 ± 2.13	**0.0019**	1.02 ± 0.89	0.4261	1.73 ± 0.53	**0.0207**	0.90 ± 0.80	0.2783	0.90 ± 0.47	0.6454	0.14 ± 0.29	0.0535	0.20 ± 0.22	0.065
**6**	1.53 ± 0.77	0.95 ± 0.91	4.68 ± 2.48	**0.0022**	7.81 ± 7.88	**0.0002**	2.25 ± 1.75	0.3512	11.50 ± 7.75	**0.0003**	2.56 ± 1.25	0.2031	12.70 ± 6.02	**0.0011**	0.70 ± 0.66	**0.0347**	3.80 ± 4.78	**0.0482**
**9**	0.24 ± 0.67	0.65 ± 4.08	2.68 ± 2.02	**0.003**	11.58 ± 3.30	**0.0047**	2.03 ± 2.43	**0.0065**	13.53 ± 5.58	**0.0011**	2.66 ± 1.00	**0.0006**	10.79 ± 5.11	**0.003**	0.23 ± 0.31	0.4834	0.90 ± 0.59	0.9807

Supplementary Table 5. Fraction of mice with protective antibody (Ab) titers; log IgG Ab titer >1.5 protective against bacteremia or >2.5 protective against lung infection following challenges with *S. pneumoniae*.

	Pnc1-TT		+mmCT		+MF59		+IC31		+Alum	
**Week**	**> log 1.5**	**> log 2.5**	**> log 1.5**	**> log 2.5**	**> log 1.5**	**> log 2.5**	**> log 1.5**	**> log 2.5**	**> log 1.5**	**> log 2.5**
**2**	0/9	0/9	4/9	0/9	5/9	0/9	4/9	0/9	1/9	0/9
**4**	1/8	0/8	8/8	7/8	7/7	5/7	3/4	5/8	7/8	0/8
**6**	2/8	1/4	8/8	7/8	7/7	7/7	7/8	6/8	7/8	2/8
**8**	5/9	3/9	8/8	8/8	9/9	9/9	8/8	7/8	5/8	1/8
**9**	5/9	3/9	8/8	8/8	9/9	9/9	8/8	8/8	6/8	0/8
